# hMG addition affects the change in progesterone level during IVF stimulation and LBR: a retrospective cohort study

**DOI:** 10.1186/s12958-023-01150-1

**Published:** 2023-12-06

**Authors:** Victoria G. Wesevich, Serin I. Seckin, Dawn A. Kelk, Amanda N. Kallen, Pinar H. Kodaman

**Affiliations:** 1 Division of Reproductive Endocrinology and Infertility Department Obstetrics, Gynecology and the Reproductive Sciences, Yale University School of Medicine, 333 Cedar Street, P.O.Box 208063, New Haven, CT 06520 USA; 2 Columbia University Fertility Center, Columbia University Medical Center, New York, NY USA; 3 Extend Fertility, New York, NY USA; 4 University of Vermont Medical Center, Burlington, VT USA

**Keywords:** Peak progesterone, Controlled ovarian stimulation, Gonadotropins

## Abstract

**Background:**

Premature progesterone (P) rise during IVF stimulation reduces endometrial receptivity and is associated with lower pregnancy rates following embryo transfer (ET), which can influence provider recommendation for fresh or frozen ET. This study aimed to determine whether change in P level between in IVF baseline and trigger (𝚫P) is predictive of pregnancy outcome following fresh ET, and whether the ratio of gonadotropins influences P rise and, as a result, clinical pregnancy outcomes: clinical pregnancy rate (CPR) and live birth rates (LBR).

**Methods:**

Retrospective cohort study at a single fertility center at an academic institution. The peak P level and 𝚫P were modeled in relation to prediction of CPR and LBR, and the ratios of hMG:rFSH were also modeled in relation to prediction of peak P level on day of trigger, 𝚫P, and CPR/LBR in a total of 291 patients undergoing fresh embryo transfer after controlled ovarian hyperstimulation-IVF (COH-IVF).

**Results:**

𝚫P correlates with CPR, with the most predictive range for success as 𝚫P 0.7–0.85 ng/mL (p = 0.005, 95% CI 0.635, 3.636; predicting CPR of 88.9%). The optimal range for peak P in regard to pregnancy outcome was 0.15–1.349 ng/mL (p = 0.01; 95% CI for coefficient in model 0.48–3.570). A multivariable logistic model for prediction of CPR and LBR using either peak or 𝚫P supported a stronger association between 𝚫P and CPR/LBR as compared to peak P. Furthermore, an hMG:rFSH ratio of > 0.6 was predictive of lowest peak P (p = 0.010, 95% CI 0.035, 0.256) and smallest 𝚫P (p = 0.012, 95% CI 0.030, 0.243) during COH-IVF cycles. Highest CPRs were observed within hMG:rFSH ratios of 0.3–0.4 [75.6% vs. 62.5% within and outside of the range, respectively, (p = 0.023, 95% CI 0.119, 1.618)]. Highest LBRs were seen within the range of 0.3–0.6 hMG:rFSH, [LBR of 55.4% vs. 41.4% (p = 0.010, 95% CI 0.176, 1.311)].

**Conclusions:**

Our data supports use of 𝚫P to best predict pregnancy rates and therefore can improve clinical decision making as to when fresh ET is most appropriate. Furthermore, we found optimal gonadotropin ratios can be considered to minimize P rise and to optimize CPR/LBR, emphasizing the importance of luteinizing hormone (LH) activity in COH-IVF cycles.

## Background

The serum progesterone (P) level on the day of trigger for final oocyte maturation in controlled ovarian hyperstimulation-in vitro fertilization (COH-IVF) cycles has been studied extensively, with the majority of evidence supporting lower implantation rates in cycles with elevated late follicular phase P levels due to asynchrony of the embryo and the endometrium [1]. Premature P elevation is postulated to negatively impact the window of implantation due to accelerated secretory transformation of the endometrium and subsequent changes in endometrial gene and protein expression [2–5]. This premature P increase can be seen in up to 38% of COH-IVF cycles despite use of gonadotropin-releasing hormone (GnRH) analogues to suppress the premature luteinizing hormone (LH) surge [6]. The deleterious effects on pregnancy outcomes due to elevated P in COH-IVF cycles with fresh embryo transfer (ET) are not found with subsequent cryopreserved embryo transfer cycles [7]. However, while a “freeze all” approach can be a useful strategy in cycles with a premature P elevation, deferral of fresh ET has the potential for detrimental psychological and financial effects for some patients. Additionally, the cryopreservation process specifically may lead to adverse neonatal and obstetric outcomes including increased rates of large for gestational age (LGA) relative to fresh ET [8]. While studies regarding premature P rise have attempted to define a threshold value above which fresh ET should be deferred, variation in established cutoff values for P and the availability of different laboratory assays complicate defining a threshold value for P above which frozen embryo transfer (FET) should be pursued.

Moreover, modifiable factors such as the choice of gonadotropin regimen may affect the progesterone rise and can be addressed prior to cycle start to minimize the risk for premature progesterone elevation. In a retrospective cohort study of over 10,000 COH-IVF cycles, Werner et al. demonstrated that cycles using gonadotropin medications with a balanced activity of both luteinizing hormone (LH) and follicle stimulating hormone (FSH) resulted in the lowest rates of premature progesterone rise. However, in this study, the relationship between P elevation and pregnancy or live birth rates was not evaluated [9], as their study looked at both FET and fresh ET COH-IVF cycles. To our knowledge, no data exists regarding the ideal ratio of human menopausal gonadotropins (hMG), which has 1:1 LH:FSH activity, and recombinant follicle stimulating hormone (rFSH), with pregnancy outcomes as an endpoint, and to simplify clinical applicability, we chose to look at hMG to rFSH ratios as opposed to LH to FSH. Thus, we sought to explore the utility of a more individualized approach via the change in serum progesterone level (𝚫P) between COH-IVF baseline and trigger during the period of COH as a determinant of fresh ET. We also sought to identify an optimal ratio of hMG:rFSH which best predicts a reduced risk of elevated peak serum progesterone levels and 𝚫P during COH-IVF cycles and pregnancy rates following fresh ET.

## Methods

A retrospective cohort study was conducted for all patients who underwent fresh ET at Yale Fertility Center from January 2017 to December 2018 (n = 291). Collected information included all demographic, laboratory, medication regimen, ultrasound, and embryologic as well as consequent pregnancy outcome data. For each COH-IVF cycle, the following variables were collected: BMI, age, infertility diagnosis, baseline P, peak P (at time of trigger), anti-Müllerian hormone (AMH), final estradiol (E2), gonadotropin medication (types and dosages), endometrial thickness, total follicle count, follicle number greater than 15 mm, number of retrieved oocytes, embryo grade/stage, number of embryos transferred, initial human chorionic gonadotropin level, gestational sac identification, fetal heart rate detection, live birth occurrence, and number, gender(s) and neonatal weight(s) of live births. The 𝚫P was calculated by subtracting the baseline P level from the peak P level within a COH-IVF cycle. Gonadotropin stimulation regimens were chosen by each patient’s primary infertility specialist, accounting for the patient’s medical history and provider preference, with the majority of cycles completed as gonadotropin-releasing hormone (GnRH) antagonist cycles. Serum P levels were measured via the Roche Cobas e411 analyzer, which measures P4 levels using enhanced chemiluminescent technology in a competition assay format. Exclusion criteria included patients using donor oocytes, gestational carriers, and patients who had preimplantation genetic testing of embryos. Logistic regression was used to model peak P and 𝚫P in relation to clinical pregnancy rate (CPR), defined as detection of a fetal heart rate, and live birth rate (LBR), controlling for age, body mass index (BMI), number of embryos transferred, follicle count and size, and endometrial thickness. Data were analyzed using the Python Statsmodels library to build a multivariable logistic regression model after standard scaling of the data. A secondary analysis was performed to identify optimal hMG:rFSH ratios in relation to 𝚫P. For this analysis, the hMG:rFSH ratio was calculated by totaling the dose of hMG over the entire ovarian stimulation cycle in relation to the total dose of rFSH. The ratio of hMG:rFSH was modeled in relation to peak P and pregnancy outcomes (CPR, LBR). This secondary analysis also controlled for age, BMI, number of embryos transferred, follicle count and size on day of trigger, and endometrial thickness. This analysis was performed using the Python Statsmodels library to build a multivariable logistic regression model after standard scaling of the data. This study was approved by the Yale University Institutional Review Board.

## Results

**Fig. 1:**
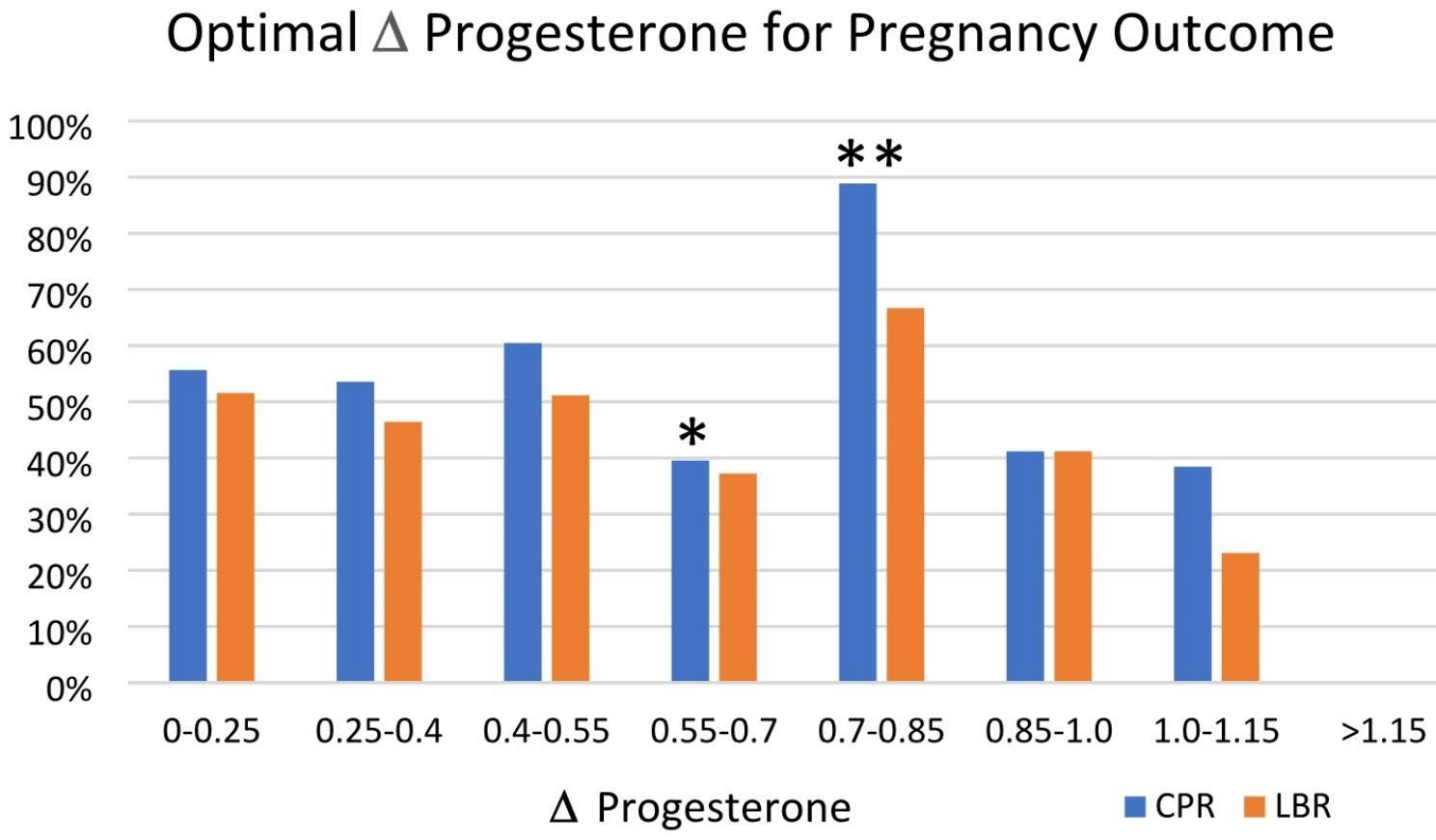
**Δ** progesterone (P) correlates with clinical pregnancy rate (CPR). The most predictive range for success: **Δ***P between 0.7–0.85* (P = 0.005; 95% CI 0.635–3.636). **Δ** P trends towards correlation with live birth rate (LBR). Association not significant, p = 0.063 95% CI -0.053-2.014. (*p < 0.05, **p < 0.005)

Our single-center cohort included a total of 291 patients, 21–42 years of age, and average BMI of 26.6 kg/m^2^ undergoing fresh IVF cycles with a GnRH antagonist protocol. Patient demographics including infertility diagnosis, age, BMI, AMH, E2 level, total number of follicles, follicles > 15 mm, and total number of oocytes retrieved is shown in Table 1. Information on embryos transferred, including the number, quality and developmental stage are shown in Table 2. The CPR and LBR in our overall dataset were 53.4%, 46.3% respectively. Our comparative models demonstrated a correlation between 𝚫P and CPR (Fig. 1 with specific values detailed in Table 3). Classifying 𝚫P as “within” versus “outside” the “optimal” ranges identified, data were stratified by increments of 0.15 ng/dL. The most optimal range for success (a CPR of 88.9%) was observed at a 𝚫P between 0.7 and 0.85 ng/mL (p = 0.005, 95% CI 0.635–3.636; Fig. 1 with specific values listed in Table 3). While no correlation was observed between 𝚫P and LBR, the relationship between 𝚫P and LBR did approach statistical significance at a 𝚫P between 0.7 and 0.85 ng/mL (p = 0.063, 95% CI 0.454–1.508).

**Table 1:** Patient Demographics

Infertility Dx	n	Age (years)	BMI (kg/m^2^)	AMH (ng/mL)	Final E2 (pg/mL)	Total Follicles	Follicles > 15 mm	Total # Oocyte Retrieved
**Male**	78	37.61 ± 3.21	25.62 ± 5.18	2.55 ± 2.71	1935 ± 791	12.13 ± 5.33	6.46 ± 6 46	10.67 ± 5.26
**Unexplained**	63	34.82 ± 3.26	25.79 ± 5.27	2.88 ± 2.02	2127 ± 865	14.30 ± 6.74	7.42 ± 3.37	12.62 ± 6.66
**Tubal**	51	34.15 ± 4.00	27.98 ± 5.87	3.33 ± 3.30	1979 ± 786	13.07 ± 6.21	6.67 ± 3.23	11.29 ± 5.90
**PCOS**	36	33.66 ± 4.38	30.13 ± 5.61	5.78 ± 3.92	2258 ± 1036	17.31 ± 6.07	8.31 ± 3.07	13.83 ± 6.42
**DOR**	31	37.61 ± 3.21	24.78 ± 4.33	0.53 ± 0.37	1362 ± 864	7.61 ± 4.26	3.97 ± 2.09	5.97 ± 3.55
*Other	18	35.17 ± 4.38	26.06 ± 5.59	2.03 ± 1.81	1839 ± 907	10.95 ± 5.32	5.56 ± 2.29	10.22 ± 7.77
**Ovulatory**	14	34.42 ± 2.90	28.4 ± 6.48	3.46 ± 2.79	2263 ± 1192	14.50 ± 6.20	7.36 ± 3.52	9.42 ± 4.52

AMH – Anti-Mullerian Hormone, E2 – estradiol, PCOS - Polycystic Ovarian Syndrome; DOR – Diminished Ovarian Reserve; *Other included:2 same sex, 1 genetic, 5 endometriosis, 3 recurrent pregnancy loss (RPL), 2 uterine factor

**Table 2:** Information on embryos transferred: average embryos transferred, quality of embryos, further categorized by cleavage stage vs blastocyst and single vs multiple embryo transfers

	Transfer Cycle Number (%)	Embryos per Transfer	Good Quality Embryo (%)	Fair Quality Embryo (%)	Poor Quality Embryo (%)
Cleavage Stage 1 ET	18 (6)	1	7 (39)	6 (33)	5 (28)
Cleavage Stage 2 + ET	41 (14)	2.3	14 (34)	18 (44)	9 (22)
Blastocyst Stage 1 ET	145 (50)	1	140 (96)	4 (3)	1 (1)
Blastocyst Stage 1 ET	87 (30)	2.1	75 (86)	10 (11)	2 (2)
**Total**	**291**	**1.5**	**236 (81)**	**38 (13)**	**17 (6)**

Embryo Quality was defined as: Cleavage stage: Good = 8-cell with Grade 1 or 1.5, Fair = 6–10 cell with Grade 2 or better, Poor = 0-5-cell or 11+ -cell Grace 2.5-3; Blastocyst Good = 2BB or better, Fair = C x 1, Poor = Cx2

**Table 3:** Change in progesterone correlates with clinical pregnancy rates

Δ Prog	CPR	p-value	CI	LBR	p- value	CI
**0-0.25**	55.67%	0.551	(-0.366-0.688)	51.55%	0.196	(-0.182-0.885)
**0.25–0.4**	53.57%	0.789	(-0.704-0.541)	46.43%	0.523	(-0.841-0.428)
**0.4–0.55**	60.47%	0.241	(-0.264-1.049)	51.16%	0.277	(-0.289-1.012)
**0.55–0.7**	39.53%*	0.048	(-1.331- -0.005)	37.21%	0.188	(-1.128-0.222)
**0.7–0.85**	88.89%**	0.005	(0.635–3.636)	66.67%	0.063	(-0.053-2.014)
**0.85-1.0**	41.18%	0.447	(-1.417-0.625)	41.18%	0.734	(-1.214-0.855)
**1.0-1.15**	38.46%	0.312	(-1.670-0.533)	23.08%	0.111	(-2.181-0.224)
**> 1.15**	0.00%	1		0.00%	1	
**Overall**	53.4%,			46.3%		

Δ P – change in progesterone level from baseline to trigger, CRP – clinical pregnancy rate, LBR – live birth rate, *p < 0.05, **p < 0.005

To determine whether peak P or 𝚫P was more predictive of birth outcomes, we used a multivariable logistic model for prediction of CPR and LBR using either peak P or 𝚫P. The coefficient in the model for peak P was 2.025 and for 𝚫P was 2.1351. Thus, 𝚫P was slightly more predictive of CPR and LBR than peak P. The optimal range for peak P in regard to pregnancy outcome was 0.15–1.349 ng/mL (p = 0.01; 95% CI for coefficient in model 0.48–3.570) and 0.7–0.85 ng/mL for 𝚫P (p = 0.005; 95% CI for coefficient in model 0.635–3.636).

With respect to the optimal hMG:rFSH ratio, secondary comparative models identified an hMG:rFSH ratio of greater than 0.6 as predictive of both lowest peak P (p = 0.010, 95% CI 0.035, 0.256) and lowest 𝚫P (p = 0.012, 95% CI 0.030, 0.243) during COH-IVF cycles. When the relationship between hMG:rFSH ratio and birth outcomes was analyzed by classifying hMG:rFSH as “within” versus “outside” the “optimal” ranges identified, the highest CPR was observed at an hMG:rFSH ratio between 0.3 and 0.4 [75.6% vs. 62.5% CPR within and outside of this range, respectively; p = 0.023, 95% CI 0.119, 1.618]; Table 4. The highest LBR was observed within the ratio range of 0.3–0.6 hMG:rFSH (LBR of 55.4% vs. 41.4%; p = 0.010, 95% CI 0.176, 1.311), Table 5.

**Table 4:** The Gonadotropin ratio range of hMG:rFSH of 0.3–0.4 was the most predictive of the optimal clinical pregnancy rate (CPR): (75.6% vs. 62.5% CPR within and outside of this range) respectively; p = 0.023, 95% CI 0.119, 1.618];

Gonadotropin Ratio	CPR Count	Total	CPR %
**rFSH only**	40	73	55%
**0-0.3**	32	73	44%
0.3–0.4*	27	40	68%
**> 0.4**	57	105	54%
**Total**	156	291	54%

*p = < 0.05

**Table 5:** The Gonadotropin ratio range of hMG:rFSH of 0.3–0.6 was the most predictive of the optimal live birth rate (LBR): (LBR of 55.4% vs. 41.4%; p = 0.010, 95% CI 0.176, 1.311)

Gonadotropin Ratio	LB Count	Total	LBR
**rFSH only**	32	73	44%
**0-0.3**	26	74	35%
0.3–0.6*	56	100	56%
**> 0.6**	21	44	48%
**Total**	135	291	46%

*p = < 0.05

## Discussion

Progesterone is essential for the development of a receptive endometrium for implantation, but high circulating progesterone levels at the end of the follicular phase in COH-IVF cycles accelerate the secretory transformation of the endometrium and negatively affect pregnancy rates [10]. An increased P level decreases chances of implantation by altering uterine-embryo synchrony. Current clinical practice focuses on a peak P level as a threshold-measure included in guidance for the recommendation for a “freeze-all” approach. There are likely some patients who are encouraged to defer fresh ET due to surpassing an established P threshold, who rather had a high baseline P and minimal progesterone rise, and may have benefited psychologically and financially from proceeding with fresh ET. Additionally, an absolute P threshold is fallible as progesterone assays vary between laboratories, and the threshold for an optimal serum P level may also vary between clinical locations as multiple threshold values have been studied in the literature. However, prior to this study, the predictive capacity of the relative change in P level from baseline to time of trigger (𝚫P) in relation to pregnancy rate was not known. Furthermore, the optimal range for pregnancy outcome in regard to peak P in our study was wide (0.15–1.35 ng/mL), emphasizing the potential weakness of using a threshold peak P as opposed to a more individualized measure which considers a patient’s baseline P level. Our results suggest that 𝚫P is more predictive than peak P. Specifically, based on our findings, a 𝚫P of 0.7–0.85 ng/mL appears to be the “ideal” change in progesterone during COH-IVF cycles, which results in optimal clinical pregnancy rates. Therefore, the 𝚫P level may have a higher utility in determining whether to proceed with fresh ET and can allow for individualized approach to decision making in these situations.

Moreover, the infertility specialist may intervene to prevent an unwanted rise in progesterone level by considering the ratio of gonadotropins used in the COH-IVF cycle. Our study highlights the importance of balanced use of hMG and rFSH, and specifically the incorporation of LH activity in COH regimens in regard to not only the subsequent progesterone rise but also pregnancy outcomes (CPR, LBR), which has not been previously described. Within the ovarian follicle, LH stimulates theca cells to produce androgens by the induction of genes involved in steroidogenesis, including cytochrome P450 CYP 17 hydroxylase and 17–20 lyase, which convert progesterone and pregnenolone to 17-hydroxylated products and androgens via the 𝚫4 and 𝚫5 pathways, respectively [11]. Further, FSH stimulates P synthesis within the granulosa cell without luteinization via up-regulation of 3β-hydroxysteroid dehydrogenase, converting pregnenolone to P [12]. Thus, a lack of LH in the late follicular phase of stimulated cycles likely allows for follicular progesterone production to exceed the limit of LH activity and contributes to a premature progesterone rise [13]. Indeed, a randomized trial comparing serum and follicular fluid levels of progesterone of patients treated with either rFSH or hMG found that the use of rFSH in the gonadotropin regimen as opposed to hMG results in higher progesterone levels, further supporting the theory that folliculogenesis in the absence of sufficient LH activity leads to premature granulosa cell luteinization [14]. Thus, the need for a balanced activity of both FSH and LH activity during COH-IVF cycles is intuitive. However, the “ideal” ratio of hMG:rFSH (a modifiable factor in COH-IVF cycles) specifically one which can achieve an ideal 𝚫P of 0.7–0.85 and result in optimal pregnancy outcomes following fresh embryo transfer, has not been determined. While one prior study has evaluated gonadotropin ratios relative to progesterone rise and identified a ratio of 0.3–0.6 as optimal, in that study, their ratio was of ‘LH activity’ to ‘FSH activity’ as opposed to a ratio of units of dosed hMG to rFSH as in the present study [9]. In this study, for ease of use clinically, we used the gonadotropin regimen ratios of hMG (with 1:1 LH:FSH activity) and rFSH dosages and we show that an hMG:rFSH ratio of < 0.6 is associated with the greatest risk of premature progesterone rise. The finding that regimens with the lowest LH activity had the highest levels of unwanted progesterone rise is consistent with the results seen in the prior study, Werner et al. Unlike the prior study, we further identified ratios which are predicative of pregnancy and live birth rates, showing that an hMG:rFSH dosing ratio of between 0.3 and 0.6 is associated with the highest LBR; a smaller range of 0.3–0.4 is additionally associated with the highest CPR, which has not been reported previously. Thus, appropriate proportional use of both hMG and rFSH, specifically incorporating LH activity, should be considered in all fresh IVF cycles in order to achieve optimal outcomes. We acknowledge the limitations of our relatively small sample size which may explain the lack of overlap between the hMG:rFSH ratio minimizing 𝚫P (> 0.6) and CPR (0.3–0.4). We emphasize that the ratio of 0.3–0.6 was predictive of LBR, which is somewhat consistent with the determined hMG:rFSH ratio of > 0.6 that best predicts a lower 𝚫P. While CPR did not overlap, it not as clinically relevant clinical as LBR. A larger number of patients is needed to clarify the relationship or exact threshold.

A strength of our study’s design is its clinical utility, as its observational design demonstrated values determined from women who ultimately chose to undergo fresh ET. Given the average age of 35 with BMI 26 and 44% had tubal or male factor infertility, our cohort represents patients with a generally good prognosis. Of note, or sample did include a broad range of infertility diagnoses including 10% having DOR and 12% having PCOS. Thus, these findings are reflective of a representative patient sample who indeed elect for fresh ET, and may serve as a guide for clinical decision making for similar eligible patients who prefer to proceed with fresh ET. Additionally, our observational design and resulting exclusion of patients who did not undergo fresh ET resulted in a more conservative threshold for progesterone level, as our levels of peak and calculated 𝚫P are likely lower than what would be seen across all patients who underwent COH-IVF cycles. There is unlikely to have been selection bias for fresh embryo transfers unique to our clinic, as our clinic performed approximately equivalent proportions of fresh and frozen embryo transfers in the time period of this study (2017–2018): 50.1% fresh ETs and 49.9% FETs.

A more robust, prospective study with an interventional design, assigning stratified ratios of gonadotropins and then having all participants proceed with fresh ET despite regardless of peak or 𝚫P, would not be feasible in a typical infertility patient population due to the potential for adverse effects on pregnancy rates.

Moreover, previous studies have demonstrated that the adverse effects of progesterone rise are of most significance for women with low and intermediate ovarian response to COH as opposed to high response; these women are considered to be particularly susceptible to the negative effects on implantation from a premature progesterone rise [15]. Our study considered the contribution of this factor in our analysis, controlling for this variation in 𝚫P as it relates to follicle and retrieved oocyte counts. Finally, while by its nature, our retrospective study does not delineate a causative relationship between 𝚫P and pregnancy outcomes, such a relationship would be further supported by gene expression studies demonstrating changes in endometrial receptivity profiles, similar to those studies comparing endometrial samples of specific progesterone thresholds in women undergoing COH-IVF cycles [2–5].

## Conclusions

In conclusion, our findings suggest that it would be advantageous to consider each patient’s change in progesterone level (𝚫P) during IVF stimulation in the decision to proceed with fresh ET versus deferral. While FET using embryos created from oocytes retrieved in the setting of premature progesterone rise appear to be successful [16, 17], fresh ET may be preferable to some patients for personal or financial reasons. Additionally, the increased rates of LGA in FET relative to fresh ET has been well established in multiple cohort studies, and further supported by sibling studies [18–20]. LGA deliveries are associated with complications including shoulder dystocia, postpartum hemorrhage (PPH) and cesarean section (CS). Recent data suggests that this outcome is most pronounced in programed (i.e. medicated) compared to natural FET cycles [21], and that programmed FET cycles are also associated with higher rates of hypertensive disorders of pregnancy, PPH, and CS [22]. Therefore, in these situations, it is important to consider strategies to avoid unnecessary delay of fresh transfers that can ultimately result in a live birth, especially given the additional cost and use of resources for cryopreservation. Our data not only support use of a patient’s 𝚫P rather than utilization of a singular progesterone threshold level to improve clinical decision making as to when fresh ET is most appropriate, but also lend support for an optimal gonadotropin ratio to minimize the rise in progesterone during COH-IVF cycles. Clinicians can start COS cycles with a hMG:rFSH ratio > 0.6 and be mindful to adjust as needed throughout the cycle, if possible.

## Data Availability

The datasets generated and analyzed during the current study are not publicly available due to preference of dataset creator but are available from the corresponding author on reasonable request.

## Abbreviations

P: Progesterone
ET: Embryo transfer
𝚫P: Delta progesterone
CPR: Clinical pregnancy rate
LBR: Live birth rate
COS: IVF-controlled ovarian hyperstimulation-IVF
hMG: Human menopausal gonadotropin
(r)FSH: (Recombinant) Follicle stimulating hormone
LH: Luteinizing hormone
GnRH: Gonadotropin-releasing hormone
LGA: Large for gestational age
FET: Frozen embryo transfer
AMH: Anti-Müllerian hormone
E2: Estradiol
BMI: Body mass index
PPH: Postpartum hemorrhage
CS: Cesarean section
